# Neonatal Spina Bifida: Clinical Profile, Management, and Maternal Characteristics in a Moroccan Tertiary Center

**DOI:** 10.7759/cureus.106431

**Published:** 2026-04-04

**Authors:** Mohammed Ech-Chebab, Inasse Lamouri, Anass Ayyad, Hanae Bahari, Sahar Messaoudi, Rim Amrani

**Affiliations:** 1 Department of Neonatology and Neonatal Intensive Care, Mohammed VI University Hospital, Oujda, MAR; 2 Faculty of Medicine and Pharmacy of Oujda, Mother and Child Health Laboratory, Mohammed First University, Oujda, MAR

**Keywords:** fenugreek exposure, folic acid deficiency, hydrocephalus, maternal risk factors, myelomeningocele, neonatal outcomes, spina bifida

## Abstract

Introduction: Spina bifida is a common neural tube defect and remains a significant public health concern in low- and middle-income countries. Data on neonatal presentation and maternal characteristics in Eastern Morocco are limited.

Methods: We conducted a retrospective descriptive study over a nine-year period in the neonatal intensive care unit of a tertiary hospital in Eastern Morocco. All neonates diagnosed with spina bifida were included. Data on clinical features, maternal characteristics, management, and outcomes were analyzed.

Results: Thirty-three neonates were included. Myelomeningocele was the most frequent type. Common complications included hydrocephalus (45%), motor deficits (30%), and orthopedic abnormalities (39%). Surgical management was performed in more than half of the cases, and some patients required ventriculoperitoneal shunting. Maternal characteristics included inadequate folic acid supplementation, consanguinity, diabetes, and reported consumption of fenugreek during pregnancy. Survival at discharge was generally high, although morbidity remained significant.

Conclusion: Neonatal spina bifida remains a significant cause of morbidity in our setting. Maternal characteristics, particularly inadequate folic acid supplementation, underline the importance of preventive strategies. The reported consumption of fenugreek during pregnancy warrants further investigation. These findings highlight the need for improved prenatal care and preventive strategies.

## Introduction

Spina bifida is one of the most common neural tube defects and remains a significant cause of neonatal morbidity and long-term disability worldwide [1]. Its incidence is higher in low- and middle-income countries, where access to prenatal diagnosis and preventive measures may be limited [2]. Despite well-established preventive strategies, particularly periconceptional folic acid supplementation, neural tube defects continue to occur [3].

Several maternal factors have been reported in the literature in relation to spina bifida, including inadequate folic acid intake, maternal diabetes, and consanguinity. In addition, exposure to certain environmental and traditional substances during pregnancy has been suggested as a potential contributing factor [4,5]. In Morocco and other Mediterranean countries, the use of fenugreek (Trigonella foenum-graecum) is common during pregnancy due to its perceived benefits, including appetite stimulation and facilitation of childbirth [6].

Previous studies from Morocco have raised concerns about a possible relationship between fenugreek consumption and neural tube defects, mainly based on pharmacovigilance data and observational reports [6]. However, clinical data supporting this relationship remain limited, particularly in neonatal populations.

In Eastern Morocco, there is a lack of comprehensive data describing the clinical profile, management, and outcomes of neonates with spina bifida, as well as the distribution of maternal characteristics in this context.

The aim of this study was to describe the clinical characteristics, management, and outcomes of neonates with spina bifida in a tertiary care center in Eastern Morocco, as well as to describe maternal characteristics and reported exposures, including fenugreek consumption during pregnancy.

## Materials and methods

This was a retrospective descriptive study conducted over a nine-year period in the neonatal intensive care unit of Mohammed VI University Hospital, a tertiary care center in Eastern Morocco. A non-probability consecutive sampling method was used. All neonates diagnosed with spina bifida and admitted during the study period were initially screened (n=35). Two cases were excluded due to incomplete or missing essential medical records. A total of 33 neonates were included in the final analysis.

Data were collected retrospectively from medical records using available clinical documentation. The variables analyzed included neonatal demographic characteristics (age at admission and sex), type and location of spina bifida, and associated malformations. Spina bifida was classified based on clinical and radiological findings into myelomeningocele and meningocele.

Maternal data included age, history of consanguinity, diabetes, folic acid supplementation during pregnancy, and reported consumption of fenugreek. Information on fenugreek consumption was based on available medical records and patient-reported history documented at admission when available. Adequate folic acid supplementation was defined as regular intake during the periconceptional period (before conception and during the first trimester), in accordance with standard recommendations. Inadequate supplementation was defined as absent or irregular intake. Information on prenatal follow-up was also collected when available.

Complications such as hydrocephalus, motor deficits, and orthopedic abnormalities were recorded. Management data included surgical intervention, timing of surgery, and the need for ventriculoperitoneal shunting.

The primary outcomes assessed were survival during hospitalization and the presence of neurological or orthopedic sequelae at discharge.

Data were analyzed using descriptive statistics. Qualitative variables were expressed as frequencies and percentages, while quantitative variables were summarized using means and ranges. No inferential statistical analysis was performed, given the descriptive design of the study. Statistical analysis was conducted using Microsoft Excel.

Ethical considerations

This retrospective study was conducted at Mohammed VI University Hospital in Oujda, Morocco, in accordance with institutional ethical standards. According to local regulations and the policies of the local Ethics Committee, formal ethical approval was waived for retrospective studies using anonymized data. Patient confidentiality was strictly maintained, and no identifiable information was included.

## Results

A total of 33 neonates diagnosed with spina bifida were included in this study. There was a slight male predominance, with a sex ratio of 1.2. The majority of patients were admitted within the first days of life.

The distribution of maternal characteristics is summarized in Table 1. A large proportion of mothers had inadequate folic acid supplementation (82%). Consanguinity was reported in 24% of cases. Reported consumption of fenugreek during pregnancy was observed in 12.1% of cases, while maternal diabetes was identified in 15.1% of cases.

**Table 1:** Maternal characteristics

Variable	n (%)
Inadequate folic acid supplementation	27 (82%)
Consanguinity	8 (24%)
Diabetes	5 (15.1%)
Fenugreek consumption	4 (12.1%)

Myelomeningocele was the most frequent type, accounting for 63.6% of cases, followed by meningocele. The lumbosacral region was the most commonly affected site. Clinical characteristics and complications are summarized in Table 2.

**Table 2:** Clinical characteristics and complications

Variable	n (%)
Myelomeningocele	21 (63.6%)
Hydrocephalus	15 (45%)
Motor deficit	10 (30%)
Orthopedic abnormalities	13 (39%)

Hydrocephalus was observed in 45% of cases. Motor deficits were present in 30% of cases, and orthopedic abnormalities, including clubfoot and limb deformities, were found in 39% of neonates.

In terms of management, surgical repair was performed in 54.5% of neonates. Ventriculoperitoneal shunting was required in 24% of cases due to associated hydrocephalus. Outcomes are summarized in Table 3.

**Table 3:** Management and outcomes VP: Ventriculoperitoneal

Variable	n (%)
Surgery	18 (54.5%)
VP shunt	8 (24%)
Survival at discharge	30 (90%)

Survival at discharge was observed in 90% of cases. However, a proportion of patients developed neurological and orthopedic sequelae requiring long-term follow-up.

## Discussion

Spina bifida remains a major public health concern, particularly in low- and middle-income countries, where preventive strategies are not consistently implemented [1,2,7]. The epidemiology of neural tube defects varies widely across regions, with higher rates reported in settings with limited access to prenatal care and folic acid supplementation [2,8].

In our study, myelomeningocele was the most frequent form, accounting for 63.6% of cases. This finding is consistent with previous studies reporting the predominance of this severe form of spina bifida [3,9]. The lumbosacral region was the most commonly affected site, in agreement with data reported in the literature [4,10].

Hydrocephalus was observed in 45% of cases in our cohort. This complication is commonly associated with spina bifida and often requires neurosurgical management [5,11]. In our series, a proportion of patients required ventriculoperitoneal shunting, reflecting the burden of associated central nervous system complications.

Maternal characteristics observed in this study are consistent with factors reported in the literature in relation to neural tube defects. A high proportion of mothers did not receive adequate folic acid supplementation (82%), which remains a key modifiable factor [3,8]. Consanguinity, observed in 24% of cases, has also been reported in relation to congenital anomalies in several studies [4,12]. Maternal diabetes, identified in 15.1% of cases, is another factor described in the literature [5,13].

One of the notable findings in our study is the reported consumption of fenugreek during pregnancy in 12.1% of cases. In Morocco and other Mediterranean countries, fenugreek (Trigonella foenum-graecum) is widely used as a traditional remedy during pregnancy [6]. Previous studies have raised concerns about a possible relationship between fenugreek exposure and neural tube defects, mainly based on pharmacovigilance data and observational reports [6]. However, given the descriptive design of our study and the absence of a control group, no inference regarding association or causality can be made. These findings should be interpreted as descriptive observations that warrant further investigation in appropriately designed studies.

A previous study conducted in the same center reported a markedly higher rate of fenugreek consumption (70%) among mothers of neonates with neural tube defects [14]. This discrepancy compared to our findings may be explained by several factors, including differences in study design, sample size, and inclusion criteria. In addition, variations in data collection methods and the retrospective nature of our study may have led to under-reporting of fenugreek consumption due to recall bias or social desirability bias.

The management of spina bifida in our setting remains challenging. More than half of the neonates underwent surgical repair, and a proportion required ventriculoperitoneal shunting. Similar findings have been reported in other studies from resource-limited settings, where delayed diagnosis and limited access to specialized care may influence outcomes [7,15]. Although survival at discharge was high, morbidity remains significant, with neurological and orthopedic sequelae requiring long-term follow-up.

This study has several limitations. Its retrospective design and single-center setting may limit the generalizability of the findings. The relatively small sample size (n=33) reduces the statistical power of the analysis. In addition, incomplete maternal data and reliance on medical records may have led to underestimation of certain maternal characteristics, particularly traditional substance use such as fenugreek, due to potential reporting bias. Finally, the absence of detailed data on folic acid dosage, compliance, and timing may limit the interpretation of supplementation adequacy.

Despite these limitations, this study provides valuable insight into the clinical profile and maternal characteristics of neonatal spina bifida in Eastern Morocco.

## Conclusions

Neonatal spina bifida remains a significant condition associated with considerable morbidity in our setting. Our findings highlight the predominance of myelomeningocele and the frequent occurrence of complications such as hydrocephalus, often requiring neurosurgical intervention. Although survival at discharge was generally high, the risk of long-term neurological and orthopedic sequelae remains important.

Maternal characteristics observed in this study, particularly inadequate folic acid supplementation, underline the importance of preventive strategies. The reported consumption of fenugreek during pregnancy in a proportion of cases warrants further investigation, given the widespread use of traditional practices in our context. These findings emphasize the need for improved prenatal care, health education, and preventive strategies to reduce the burden of neural tube defects in our region.
